# The ‘vulnerability code’: Is cell identity the architect of its own decay?

**DOI:** 10.1002/ctm2.70555

**Published:** 2025-12-15

**Authors:** Dongsheng Bai, Zhenkun Cao, Chenxu Zhu

**Affiliations:** ^1^ New York Genome Center New York New York USA; ^2^ Department of Systems and Computational Biomedicine Institute for Computational Biomedicine Weill Cornell Medicine New York New York USA; ^3^ Physiology, Biophysics and Systems Biology Graduate Program Weill Cornell Medicine New York New York USA; ^4^ Memorial Sloan Kettering Cancer Center New York New York USA

1

The establishment and maintenance of cellular identity depend on two fundamental yet historically separately studied mechanisms: DNA repair machineries that safeguard genome fidelity, and epigenetic programs that regulate cell‐type‐specific gene expression patterns.^1^ Research on DNA damage repair has primarily emphasised the molecular pathways and kinetics of the DNA damage response.^2^ Conversely, investigations on epigenetics have focused on how cells establish and sustain their transcriptional memory.^3^ This disciplinary separation has created a blind spot: while we understand how the hardware (genome integrity) fails and how the software (epigenetic regulation) becomes compromised, the question of whether and how the former impairs the latter remains unresolved.

Recent research indicates that these two processes are interconnected. The link between them is recognised through the understanding that DNA damage constitutes an effective ‘toxic modification’ that influences gene regulation. When cells experience genotoxic stress, chromatin temporarily relaxes to permit the access of repair factors to DNA; this process involves the phosphorylation of histone H2AX and the recruitment of chromatin remodellers.^4^ Ideally, these changes are transient and are reversed once the damage has been repaired. However, some modifications may not be fully reversed, leaving persistent epigenetic ‘scars’ that affect gene expression long after the repair process.^2^ Conversely, the existing epigenetic context influences the genome's vulnerability: heterochromatin regions tend to undergo slower repair, while active regions such as promoters and enhancers are more accessible but also more prone to damage caused by transcriptional activity or toxins.^5^ This results in a complex, system‐wide challenge: the concept that chromatin modifiers are repurposed for DNA repair suggests that the balance of cell survival and proper functioning involves an ongoing conflict at the molecular level. This bidirectional relationship constitutes a ‘systems level’ issue: the ‘*relocalization of chromatin modifiers*’ theory proposes that the machinery used for repairing DNA breaks is frequently borrowed from the epigenetic maintenance system, leading to a direct conflict between the maintenance of cell survival and the preservation of cellular function.^6^

These findings challenge the long‐held view in the field that DNA damage occurs randomly primarily due to thermodynamic noise and environmental factors. If DNA damage hotspots can be precisely identified, the trajectory of cellular decline may become more predictable, as the initial regulatory failure can be specifically targeted. The susceptibility of organs to DNA damage varies considerably among mammals: the most vulnerable are energy‐dense tissues such as the brain, owing to their reliance on abundant redox‐active compounds. Indeed, within the nervous system, long‐lived cells tend to accumulate damage over extended periods, highlighting the importance of these mechanisms in neural aging and pathology. Recent studies have demonstrated significant correlations between elevated DNA damage, the gradual decline of the epigenetic landscape and the development of age‐related neurodegenerative disorders,^7^, ^8^ indicating that an imbalance between DNA repair mechanisms and epigenetic stability may contribute to neuronal aging and disease progression. This creates a major technical challenge: precisely identifying damage hotspots across different cell types within complex, heterogeneous tissues.

To bridge this gap, we developed Paired‐Damage‐seq, a method for single‐cell parallel analysis of oxidative and single‐strand DNA breaks alongside the transcriptome.^9^ We first benchmarked Paired‐Damage‐seq in a cultured cell line: the specificity of damage detection was validated against previously established bulk assays, and the sensitivity was evaluated using publicly available single‐cell datasets (of transcriptome and epigenome). Additionally, analysis of stress‐treated cells showed coordinated changes between DNA damage accumulation and epigenomic shifts, highlighting the method's ability to detect both baseline and stress‐induced patterns. By applying this technology to the mouse cerebral cortex, we confirmed that DNA damage hotspots are not randomly distributed: instead, regulatory hotspots, including nucleosome‐depleted regions, bear the bulk of the burden. As expected, Paired‐Damage‐seq demonstrated that distinct cell populations (e.g., neurons versus glia) exhibit unique vulnerability profiles associated with their specific cellular functions. For example, neuron cells tend to accumulate damage in synaptic genes, whereas glial cell‐specific DNA damage hotspots are enriched in metabolic regulatory regions. This indicates that the ‘cost’ of cellular identity constitutes a particular vulnerability to damage within the specific genomic elements that establish that identity.

The identification of DNA damage hotspots offers valuable mechanistic insights: DNA damage repair not only maintains the structural integrity of the genome but also preserves epigenetic stability. Our findings indicate that these damage hotspots may serve as early markers of epigenetic changes, as they tend to lose epigenetic memory faster than other genomic regions (the ‘vulnerability code’). This process potentially results in a ‘scarring’ effect on the chromatin landscape. When repair factors are repeatedly recruited to the same hotspots, the local epigenetic states (such as histone and DNA modifications) may not be perfectly restored (Figure 1). This aligns with the ‘*Information Theory of Aging*’, which proposes that the noise produced by DNA repair processes gradually damages epigenetic memory, causing a decline in cellular identity.^10^ Consequently, a map of DNA damage can serve as a useful prediction tool for the cell's future state. By locating ongoing genomic breaks, it becomes possible to anticipate which genes might soon lose their regulatory control. This shifts the perspective on aging from a stochastic to a more deterministic process; by identifying key hotspots, we can improve predictions of future epigenomic changes.

**FIGURE 1:**
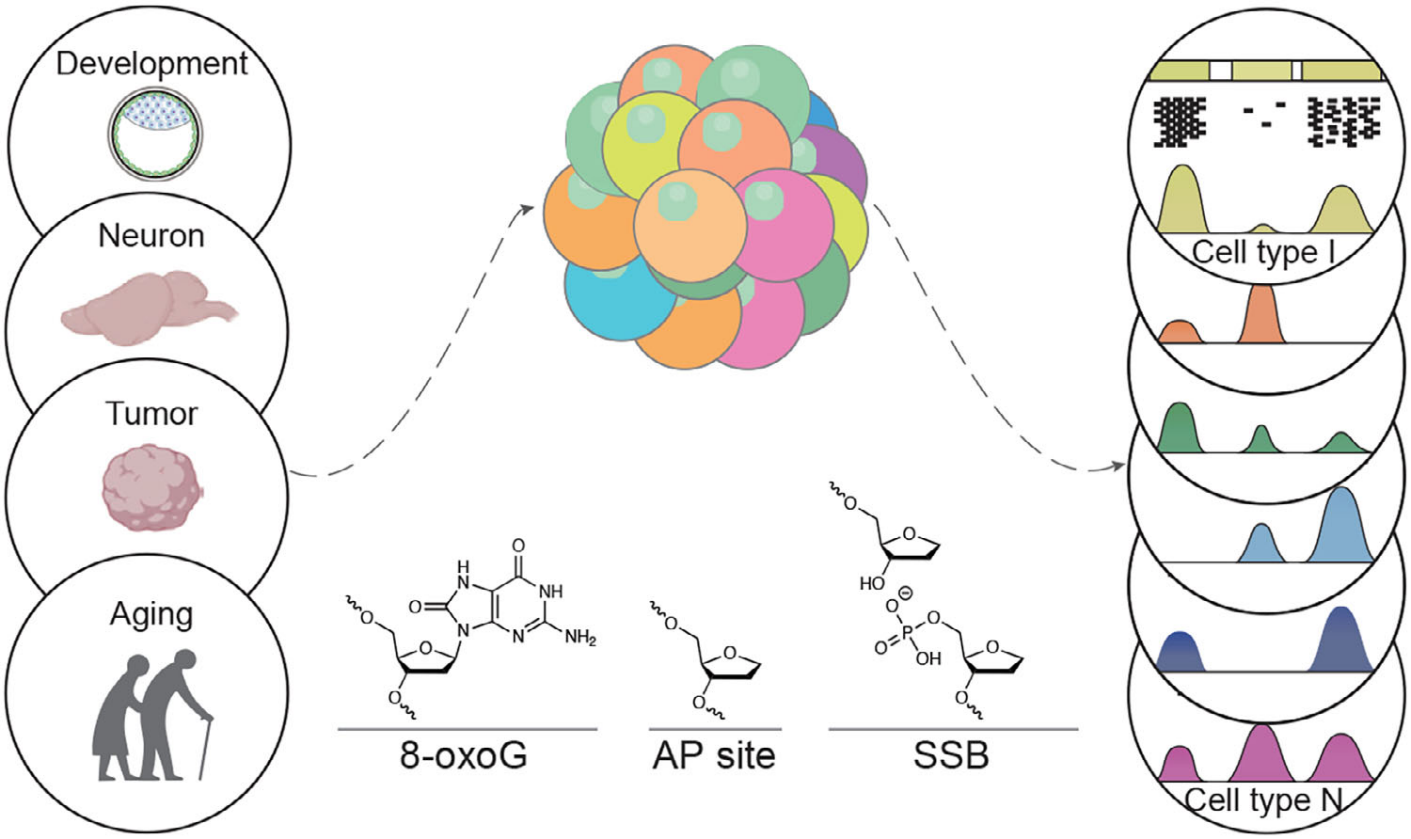
The ‘vulnerability code’ of the genome. Cell‐type‐specific epigenetic profiles are essential to the processes of development, aging, and disease. The pre‐existing epigenetic states predispose the genome to damage related to metabolic processes, such as oxidative damage and single‐strand DNA breaks, which may subsequently lead to the erosion of epigenetic memory over time.

This shift from ‘stochastic’ to ‘deterministic’ has immediate translational implications for the diagnosis and treatment of age‐related diseases. By identifying these ‘fragile’ regulatory regions, we can detect the earliest signs of cellular decline, potentially years before pathology manifests or global epigenetic changes become noticeable. Furthermore, this understanding opens new avenues for therapeutic intervention. Current therapeutic approaches often depend on broad‐spectrum antioxidants or general epigenetic modulators,^1^, ^2^ which may lack sufficient specificity to achieve optimal efficacy. Future interventions could focus on reinforcing local epigenetic states (such as chromatin accessibility) at the specific DNA damage hotspots, rather than attempting to globally protect the entire genome. Ultimately, by integrating the study of DNA damage and epigenetics, we are entering a new era of precision medicine. By mapping the process of cellular identity erosion, we can potentially intervene to arrest or even reverse it.

## CONFLICT OF INTEREST STATEMENT

C.Z. and D.B. are listed as inventors of a provisional patent application related to Paired‐Damage‐seq.
